# A Plea for Principles and Conservatism in the Treatment of Diseases Peculiar to Females, Embracing the Report of a Case of Superinduced Pseudo-Cyesis, with Results

**Published:** 1876-07

**Authors:** Wm. Abram Love

**Affiliations:** Professor of Physiology in the Atlanta Medical College, Atlanta, Ga.


ATLANTA

MEDICAL AND SURGICAL JOURNAL

VOL. XIV.] JULY—1876. [No. 4

### Original Communications.

### A PLEA FOR PRINCIPLES AND CONSERVATISM IN THE TREATMENT OF DISEASES PECULIAR TO FEMALES, EMBRACING THE REPORT OF A CASE OF SUPERINDUCED PSEUDO-CYESIS, WITH RESULTS.

BY WM. ABRAM LOVE, M.D.,
Professor of Physiology in the Atlanta Medical College, Atlanta, Ga.

The following case is reported for the purpose of showing—not only the tortures to which some unfortunate suffering females are subjected by a class of professional itinerants who make pretensions to special work—but for the purpose of showing the claims of such itinerants to the confidence of the people. Aside from this, the case presents some points of interest to the profession, to which I shall allude in the further progress of this article.

About the 25th of October, 1872, a gentleman called at my office and stated to me that his wife's medical attendant had been compelled to leave the city for a season—that before his return her approaching accouchement would come off; and that on that occasion they desired my professional services; and that she would be pleased to make my acquaintance at the earliest time it might comport with my convenience to call.

On the following day I made my first visit. Conducted in, I found a lady resting on a couch—a frail delicate form, much reduced in flesh; too weak to rise to meet me, and exhibiting evidences of having been a great sufferer; yet withal she seemed cheerful and hopeful. She was a lady of refinement, of superior intellect, education and accomplishments; had devoted much of her time to literary pursuits, and by reputation as a writer I had known her. My call being rather social than professional, no inquiry into her condition was instituted, any further than to inform myself as to the probable time of her expected confinement—which was near at hand—and to ascertain if due preparation had been made. Finding that everything had been anticipated, I requested her to give me timely notice when my services should be needed, and departed.

On the evening of the 31st of October I was summoned in haste by her husband, who informed me that she had been suffering to some extent since a little after midnight, and that her symptoms were of such a nature as to require my immediate attention.

Reaching her bedside, she informed me that her pains had commenced about one or two o'clock in the morning, and had continued until the then present; that at daylight she found some discharge from the uterus of a sanguineous character, but inasmuch as it did not amount to much more than an ordinary menstrual flow, she did not deem it necessary to disturb me; that the flow had somewhat increased during the day, and that she had passed one or two blood-clots during the afternoon; that her pains had been paroxysmal, but not much more severe than they had frequently been during her monthy periods. While the pains were of very little moment, as labor pains, the hæmorrhagic discharge in the outset of labor was a matter of more moment; and I therefore, to satisfy myself as to her condition, proposed an examination.

My feelings may be better imagined than described, when on reaching the os uteri, I found it patulous—the neck not obliterated, but its walls thickened—the whole organ movable but hypertrophied—in all respects, so far as the sense of touch would reveal, suggesting the idea of a *sub-involuted uterus.* The bowels were terribly distended, so much so that their size would justify one in surmising a case of amniotic dropsy. A more rigid examination, however, revealed the *phantom* nature of the case. My facial expressions aroused the fears of my patient, for in one and the same breath she anxiously inquired if anything was wrong; if she was in labor, and how long before it would terminate.

My position was one of no little embarrassment. To equivocate would be to deceive; to hesitate would be to increase the anxiety and fears of my patient; and to come out candidly and tell her plainly *that she was not only not in labor, but not pregnant,* might possibly, under all the circumstances, not be for the best. But having long since established a rule to deal candidly with my patients, I gave her frankly my opinion, that she was not only not in labor, but not pregnant. I can never forget the earnestness with which she appealed to me to know if I was convinced of the correctness of my opinion, and candid in its expression. When I told her that I was both correct and candid, she replied: "Then my medical attendant has been wofully ignorant of my condition, or he has taken advantage of that unfortunate condition for the base purpose of his own professional aggrandizement, and either being true, I want never to see him, and shall never speak to him again." All efforts at reconciling her proved futile; her mortification and indignation were unbounded, and before the history of the case is concluded, the reader will think with the writer, that it was not without cause.

Finding some increase of the menorrhagia, for the purpose of holding the flow in check and producing quiet, I prescribed pot. brom. — morphia sulph. — ext. ergot fl.; to be repeated *pro re nata,* and left the case for the night.

On the following morning I was called to see her at an early hour, but more because of the mental than the physical condition of the patient. The remedies directed the night before had controlled the excessive discharge, and there was little physical suffering. The lady stated to me that if there was the possibility of a mistake as to her condition, or if any further investigation was necessary to decide absolutely the question as to her pregnancy, she desired it made, and the question settled absolutely; and furthermore, she desired to know definitely the character of the disease, or diseases, from which she had suffered so much.

For the purpose of verifying my own opinion as expressed, and with the further intention of convincing her of the nonexistence of pregnancy, the speculum and sound were brought into requisition; the entire cavity of the uterus sounded (the measurements taken three and a half inches from external os to fundus), and there remained no longer in her own mind the least doubt on the subject.

This confirmed her in her irreconcileableness to her medical attendant, and increased her mortification and indignation. She earnestly requested that I should take charge of her case, and endeavor to restore her health. Advising her to calm quietude until she passed her period and was in a condition for a more thorough investigation of her case, by which time I would be able to get a better insight into its duration and history, I left her, continuing treatment.

The history of her case, which I obtained from time to time, I at a subsequent period requested her—she being a ready writer—to give me from her own pen, with permission to use should I desire to do so. That history will more properly come in at this place in the general history of the case, and I insert it as written by herself, taking the liberty of adding in such [parenthetic] notes or words as may seem necessary to render it more complete or definite. The circumstances under which the article was written will doubtless explain, and be a sufficient excuse for the somewhat caustic character of the production:

"*Dr. Love:*

Dear Sir—Nothing but your repeated requests, and my confidence in you as my physician, together with the sacredness of your obligation to hold from publicity the names of your patients, could ever induce me to commit to paper that which I would like to bury in the oblivion of the past. If, however, you find in the history of my case anything *apropos* to scientific investigation, in the amelioration of the diseases of my own sex, by the exposure of quackery, or the abuse of theory, I shall not regret that by my sad experience others have been saved the many things I have suffered at the hands of many physicians.

To be brief, I have been an invalid for nearly twenty-two years. My ill health dates from the birth of my only child, which occurred in my sixteenth year. Six weeks after my accouchement I performed a journey of one hundred and fifty miles, in private conveyance, over a rough and broken country. This, together with severe labor and other untoward circumstances, left my health a wreck. For a series of years I have suffered with leucorrhœa; during which time I applied to various physicians for relief. Each made his own diagnosis, and administered remedies accordingly. Some of them attributed my low state of health to vaginal relaxation, and endeavored to build up my system by tonics and astringent washes. Others administered innumerable preparations of spiced beverages, etc., all of which aggravated my malady and increased my nervous irritation at every monthly period, leaving me more enervated and fluor albus greater.

Turning from these, I submitted my case to another physician. Without any examination, he inferred from the character of the discharge that the disease was located in the uterus, and he subjected me to repeated blisterings over the region of that organ, and counter-irritants on the small of the back, such as croton oil and tartar emetic. Sometimes I was allowed to intermit these with "strengthening plasters." The long continuation of this disease gave me a dragging sensation in my loins, and he inferred that the uterus was prolapsed, and gave me, or rather applied, a glass pessary. Every variety of these were brought into requisition. I wore elastic balls and rings, and as my physician pretended to be of the progressive school, I had the benefit of every invention in the treatment. I suffered this character of practice for years, until my complaint and agony became so great that, discontinuing the use of these, and following the advice of my physician, I adopted a "Banning's Brace." This was very painful, as it pressed upon the very spot where soreness and tenderness existed. A variety of patents were used which have escaped my memory, and all of which I recur to with horror.

Exhausted in patience and physical strength, I left the locality in which I resided, for the invigorating air of higher latitudes, determined to appeal no more to *medical skill* for relief, but to rely on the health-giving properties of mineral waters. The Indian Spring, Gordon, Catoosa, and Saratoga were my resorts; none of which proved at all efficacious. The habit of physicing was confirmed, and in my desperation I sought the advice of one of the most prominent physicians of the South. He made an examination by speculum (the first that had ever been used in my treatment), and ascertained that I had been suffering with ulceration, hypertrophy, and induration of the uterus. He assured me that no prolapsus existed, and that all the tortures I had undergone were but a waste of vitality. I was much overjoyed to hear a new name for my disease, and deeply impressed with his technical lore, I had nothing to challenge my credulity, and cheerfully prepared myself for the ordeal that was to eventuate my cure in the space of five or six months. Cauterizations from ten to fourteen days apart failed to give me relief, and when I remonstrated I was commended to the gracious hand of time, with the statement that there might be some inflammation in the fundus of the organ, but that it was an innovation upon science to pretend to reach or cure any inflammation in a locality which it would be instant death to medicate. As I was trying to avoid such a catastrophe, and had no desire for a conflict with science at the expense of my life, I resigned myself to my fate, as one of the misfortunes of my sex.

Although I had intervals of great discomfort, I nevertheless maintained my energy and indomitable spirit. During three years in Virginia [She was the wife of a gallant officer in the Confederate army, and like many Southern ladies similarly related, continued near her husband during the military campaigns], whilst participating in the misfortunes of the war, I was under treatment from a homœopathic. My experience with the sugar pellets was too comical for so grave an article as this must be; therefore I desist.

My health declined during the war, and I became the patient, at different times, of several army physicians; but the irregularity of their attention eventuated in harm rather than benefit, and thus I drifted along, determined to rise superior to my almost constitutional disease. Being of a naturally buoyant spirit, and associated with the pleasures of youthful society, I unfortunately so far forgot myself as to conceive the idea of learning to skate. The many *ups and downs*, in the acquisition of this accomplishment, brought on me a spell of acute inflammation of the womb, from which I suffered intensely for four months. My physician resolved nearly all the ills of his female patients into "hysteria" or "pregnancy,"

and I fell a victim to such palliatives as assafœtida and iron, instead of checks to my painful disease. I had injured myself by repeated falls, and the inflammation increased the hypertrophy and induration. It was very many months before I received any direct remedy for my affliction. I was then put on bromide of potassium; the posterior lip of the womb was incised, which, exuding but little blood, there was then begun a process of leeching, to relieve the hypertrophy and induration. I scarcely was free from the bleeding of one operation, before I had to submit to another, and in every instance it gave me high fever and excruciating pains in the head, which lasted for days. I was under the treatment of this physician from November until the following September, at which time I left, pale, emaciated, and bed-ridden, and came to Atlanta.

For awhile the idea of a sojourn at a water-cure establishment was entertained, but I recalled to mind a two months subjection to bread and water at one of the most prominent of these, on the continent, and abandoned myself to despair.

During these hours of deep, deep despondency, I was visited by a physician who professed to be "fifty years in advance of the profession," and informed me of a discovery he had made, unknown to the science of anatomy, and which would shortly be given to the world in a volume of five hundred pages. He stated that anatomy was at fault in its estimate of the depth of the uterus in living subjects, because her conclusions were formed from the examination of post mortem cases, where the organ, by its contractile nature, misled the deductions of science. He said that for forty-three years of successful and lucrative practice, he had established to his own satisfaction the depth of the uterus to be *ten inches* [italics parenthetic], and always conformed to this theory. He said that the imperfect cures effected by other physicians was due to the misapprehension of this fact. He exhibited to me an instrument of his own invention [a grooved sound, the groove of which was, for use, filled with powdered nitrate of silver], discolored ten inches, said to have been caused by frequent penetrations to that depth by what he termed "thorough treatments." He explained how this polished shaft could deposit the healing grains with almost instinctive life. I tried to impress upon him that mine was a chronic case, but his power surmounted all my objections, as he expatiated on the wonderful cures he had made, and my faith increased as he led me beyond the pale of reason. I was enthused with the prospect of health and the display of his miraculous power. I was to be cured without any of those nauseous drugs, and as Shakespeare says, could " throw physic to the dogs." Thus I became his *patient* in more senses than one, as the sequel will show. For about three weeks I was a martyr to "theory," from ineffectual attempts to pass the steel sound to the required depth of ten inches,* which obstacle was attributed to a spasmodic action, the result of long inflammation. At last the object was accomplished, the wire [sound] withdrawn, and one of similar size and length [the grooved sound] loaded with caustic introduced and discharged. These operations were succeeded by most excruciating pains and violent cramps in the diaphragm, stomach, and bowels, a deathly sinking sickness, all necessitating immediate application of bags of hot salt,

---

The "itinerant" here alluded to created quite a sensation in this city a few years ago, by claiming to have demonstrated that the normal uterus was from nine to ten inches long, and that he could introduce a sound, when there was no malposition of the organ, from seven to nine inches from the external os. I was permitted to witness this remarkable feat in two cases: in one he introduced the sound to the depth of eight inches, and the other nine inches from the os externum, by accurate measurement. At first I was undecided as to whether he perforated the fundus of the uterus with his sound, or passed it through the fallopian tube; I was, however, in the last case able to satisfy myself that he passed the sound through the right fallopian tube. This case was a lady greatly emaciated, with abdominal walls so thin and relaxed that I could readily place my fingers above the fundus of the womb, and in this way was able to detect the sound passing through the fallopian tube, and in this instance to the depth of nine inches from the os externum. In both cases the shock was alarming, to me at least; but the Doctor evinced no uneasiness, as he said it always occurred after such treatment. In one case the shock from the introduction of the sound loaded with the nitrate of silver was as great and as instantaneous as if a pistol ball had passed through the abdominal cavity—the lady presenting the prominent symptoms of such injury, as great palor, excruciating pain, feeble and frequent pulse, vomiting, etc. These symptoms gradually subsided, under large doses of opium and brandy, with bags of hot salt to the abdomen. In an hour and a half or two hours she was comparatively comfortable. He claimed never to have killed any one by his treatment, as he called it. I, however, have doubts upon the subject.—ED.

excessive draughts of whiskey or brandy, and the use of elixir of opium or morphine in herculean doses, and these to be followed again by mustard poultices and fly blisters, with the internal administration of calomel to battle the inflammation. Sometimes the pain in the head would be intolerable; at other times the nausea and vomiting was terrible to endure. The free use of whisky and elixir of opium generally preceded the treatment. Every few days these operations would be repeated, to be followed by days of suffering and confinement to bed. I think I began to submit to this practitioner the last of September, and by the last of November I had received what he termed six "thorough treatments." This practice was intermitted in my case by the absence of the physician [?] and resumed again in February. The same obstacles that met the operation in the outset, viz: contraction of the channel of the uterus, beset him again, and after repeated efforts the organ yielded *in some way* to the satisfaction of theory. I was considered a *good subject* for experiment, and was requested to allow a physician to witness the test of his theory. I replied, "If it be for the advancement of science, and the physician a gentlemen, I urge no objection."

Three unsuccessful experiments attended these interviews, the last of which produced a discharge of blood, which continued profusely for three weeks; so great was the depletion that—as I now understand—I missed my monthly visitations for three periods. I was then declared *enceinte.* The use of the instrument was discontinued, but a new order of practice ensued. In June, about the last of the month, my periods returned. I was then placed in bed, tamponed for six consecutive days and nights, and enveloped in ice cloths. The first twenty-four hours I took from the hands of this physician one vial of elixir of opium, several doses of morphine, in addition to repeated enemas of laudanum. The tamponing irritated the rectum, occasioning internal hemorrhoids and bleeding. The catheter accompanied these *precautionary measures*, giving me such intense pain that I did not recover from its effects for weeks. You cannot imagine how I suffered; my head was almost bursting with pain; my stomach was so nauseated as to keep up a water brash for two weeks, and my intense thirst not even allowed to be gratified with ice, as it was said to excite the womb by its ejection. When I would plead for a discontinuance of this treatment he would gravely lecture me on the immorality of self-abortion. It was fully six weeks before I was able to sit up with any comfort, or to take a step in my room, and I had not been able, during a portion of *this performance,* to speak audibly my wants. Scarcely had I been enabled to recover sufficiently to sit up, before another *regular period* returned, quite painful, but not so profuse. On this occasion the same remedies were applied, but not requiring so great an effort, nor so great a sacrifice of time and *vigilance* of the physician. This left me with a dreadful cough, brought about by the ice cloths and baths to which I was subjected.

By the month of August my digestion was so much impaired that I could not gratify any desire for food without subjecting myself to terrible cramps and spells of nausea, much to the chagrin of my physician, as it seldom failed to bring about action in the uterus, or small streaks of blood from the stomach. I was never permitted to bathe my feet in warm water, lest the same result should follow.

Fortunately for my life the physician who had so persistently followed his *one idea* was called away for a time. I was so nervous that I had determined to resort to warm baths to procure sleep at night. Their effects were salutary. I had been using them about two weeks when I sent for you, and feel certain that they aided in restoring the long contravened laws of nature. This physician had me under his care for nearly one year, and must have averaged an *examination* per week from the time I first became his patient. A day or two previous to his departure he made his last examination, which he stated was perfectly satisfactory to himself, and described to me the appearance of the womb when near parturition; stating that all my fears of a diseased uterus would in a few weeks be dissipated by accouchement, and challenged the whole medical fraternity to dispute a point substantiated by an experience, as he said, of forty years in the specific diseases of females.

You know my condition when you first met me, and that in addition to the long years of disease which had produced abnormal organism, you have had to contend with the imprudences of quackery. These you have relieved, and I desire to express my gratitude to you for the same, and to say in conclusion that no purer gratitude—no warmer sympathy and love will ever cheerish your memory—than that which comes from the *time-worn invalid's chair.*" * * * *

Such is the history of the case, penned by the hand of the patient herself, without aid or suggestion from myself or any one else, save the simple request that she write it and place it at my disposal for publication should I report the case. It is replete with melancholy interest, and, though lengthy, I make no apology for giving it in full, save for the last paragraph, with its personal allusions. This would not have been inserted but for the fact that it contained a very pertinent allusion to the results of treatment in the progress of the case.*

With this I return to the pathological condition of the patient. That the case was one of *pseuda-cyesis* there could be no doubt. Auscultation gave no fœtal heart sound; percussion gave a tympanitic response. Tactile examination, while it revealed the existence of a uterus somewhat enlarged in body, neck and mouth, was still movable, and through the thin though distended walls of the bowels its outlines could be traced, revealing a size by no means commensurate with the professed period of gestation. The sound passed into the cavity, which it entered without obstruction, showed that cavity unoccupied. The abdominal distension from gas, the result of indigestion—the passage of that gas ever and anon from one fold or valve of the bowels to another, conveyed to the mind, as it often does, a sensation assimilating and often mistaken for *quickening* and *fœtal motion*—were the only points in the case resembling pregnancy. These had given rise to the *phantom* that led to such disappointment.

At my first visit she was but passing through her menstrual period. Subsequent examinations, made at a proper time, revealed abnormal conditions of the uterus; the walls of the organ were thickened, neck and body, partially filling up the pelvis; the mouth was patulous, presenting a ring around the external os from the turning out of the lining membrane of of the neck, a sort of ectropion, so often confounded with ulceration; the sound revealed abnormal depth, and photo-

* Following this case, three other ladies, who had been under the *charge* of this "itinerant," have fallen under my care, but none so severe, and all have made good their recovery.

graphing the cavity of the uterus, demonstrated the existence of ulceration in the fundus, and in the left lateral half of the upper posterior portion. The sound readily awaked contractions of the diaphragm, stomach and intestines—reflex—whenever this portion of the cavity was touched with the instrument. The mouth and vaginal portion of the neck of the uterus presented numerous traces of former ulcerations, scarifications, etc.; but were, save the ectropion, vascular engorgement, and hypertrophy, not otherwise diseased. The vagina was healthy; the ovaries were both tender, particularly the left, and the latter enlarged. So much for the exclusively gynæcological view of the case. For over twenty years she had been a sufferer, and as she says her local or uterine disease had been treated as a *local disease*, and the cause of all her ill health for as many years, without eventuating in a cure. The last medical attendant professed to cure by what he called "thorough treatments"—by local applications exclusively; he eschewed internal remedies, throwed physic to the dogs, and with it the fundamental principles, ignoring physiology, pathology, therapeutia, and claiming that anatomy was false in its teachings.

This lady had been an invalid for a score of years; her general health a wreck, yet withal no efforts were made to correct the many pathological conditions and morbid associations at work throughout her entire organism, tending to the complete destruction of that organism; and the idea is too prevalent to-day, in the treatment of diseases peculiar to the sex, that a woman consists of a womb and two ovaries, and beyond these it is unnecessary to give attention. Take this case, for example, with its preceding twelve months history and treatment. What had been done save the inhuman torturous application of caustics, with such things as might be necessary to battle against the effects of such applications? This, too, in the name of science! Verily it brings to mind the last exclamation of Madame Roland, as she mounts the scaffold and casts her eyes upon the statue of liberty: "O liberty, liberty! the sins that have been committed in thy name!"

Let us pass on with a further examination into the general pathological conditions. First, the general health of the patient was a complete wreck. Her digestion was seriously impaired. Food taken into the stomach was taken without relish, and was either rejected, or produced nausea and vomiting, or disagreed with the stomach and bowels if retained; a general torpor in the digestive apparatus presented itself; the bowels were slow to act, often constipated; at other times the reverse. The absorption of nutritive material was but partial—hence the blood tissue was impoverished, anæmic. The general circulation was weak, the portal torpid, and the capillary very imperfect; the skin dry and inactive as a consequence. The tissue-making material was wanting, and assimilation and disintegration went on slowly. The kidneys acted scantily; the urine was high colored, sp. gr. 1.024 heavily charged with octahedral crystals of oxalate of lime, and disintegrated epithelia from kidneys and bladder; the muscular tissue reduced, and showing great want of strength; much depending upon want of nutrition and exercise. The liver was inactive, as evinced by the yellowness of skin and the saffron hue of the soft palate. The entire nerve system very much depressed from want of proper disintegration and elimination, as we find in cases to which the term *cholesterœmia* has been applied, required relief from these dead elements—from the smoke and ashes, as it were, of the tissues burned out in their slow vital combustion—this half living half dying condition, which had obtained for so long a time.

These pathological conditions pointed plainly to the therapeutiæ in the case—not exclusive and local, but general and special, and each indication was met in such way as seemed to promise most in correcting these irregularities.

The disease in the internal cavity of the uterus was relieved by the local application of a solution of iodine and bromine in glycerine, made direct to the diseased fundus, after dilating the neck. These were repeated at intervals of from five to seven days, for about two months, when the ulcerative and inflammatory action was found to have given away entirely. The hypertrophy and induration was treated by taking advantage of the osmotic action of remedies—a weak solution of bromide of potash, in pure glycerine (to which was sometimes added morphia and atropia in small sized doses, but required to be used with even more caution and in less doses than by the stomach) was used as a local application to the mouth and neck of the uterus, applied on cotton floss tampons and changed every eight to twelve hours. This seemed to drain the vessels of the neck and walls of the uterus, and by its osmotic action medicate the organ regularly and continuously. Each change of the tampon was attended with free irrigation of the parts, with hot, tepid or cold water—using the one or the other, as seemed most agreeable to the patient. This constituted the local treatment.

The conditions given above indicated a cautious but not less vigorous and decided constitutional treatment, and many indications were to be met and fulfilled at one and the same time. The most prominent of these seemed to be to clear the deck for action; to remove the dead elements from the system; to clear away the rubbish and rebuild. First, then, this condition of cholesteræmia was removed by minute doses of calomel thoroughly triturated with subnit. bismuth, oxalate of cerium, and sugar of milk, and given every two or thee hours until the alvine evacuations indicated hepatic action, keeping up the action by saline waters or saline solutions. largely diluted and freely used. This cholagogue treatment was repeated as occasion should require, with care not to make a mercurial impress upon the system and further break down the wreck. The free use of the saline waters, or instead, plain water or lemonade, and such like drinks, served to act as a diuretic, to wash out the blood tissue and carry away the dead waters, as it were, and give place to more life-like fluids. To prepare these the materials must be supplied; her appetite and digestion must be built up. For this purpose resort was had to bitter tonics, wine and pepsin, modifying and changing their form, flavor or taste from time to time; catering to a stomach made fastidious by long-continued mal-action, but pressing forward on the main line, fortifying at each advance step with a more and more generous diet, as nature could use to advantage the tissue-building material placed at her disposal.

Another important aid in pursuing this plan of treatment was baths and friction, followed by exercise as the patient gained strength. Warm, cold or tepid water, sponge, plunge or douche, with crash towel, flesh-brush, or friction with the hand, were brought into requisition to great advantage in the treatment of the case; set the emunctories of the system to work, and made room for better material, with which the assimilative powers are supplied to work it up into tissue.

The over-distended bowels were placed under the gentle pressure of an elastic bandage, to support to some extent the intestinal and abdominal walls against the gaseous contents, and aid them in regaining their tone, upon the principle, if you please, of the tumefaction bandage. Two remedies were, with all the catering changes of prescription, used throughout the treatment: the one, nux vomica in some form, for its well-known nerve stimulant and tonic effects; the other, ergot. The latter was continuously administered, at times to the extent of bringing uterine contraction, for the purpose of arousing action and restoring tone to the uterine tissue, and overcoming the vascular engorgement, through its known influence over the vaso-motor system. Belladonna was used as the condition of the patient would seem to demand, for the purpose of overcoming a tendency to constipation, or to *flush* the capillaries when the flow through them would fall below the healthy standard for any considerable time.

Watching my patient carefully, keeping her forces as nearly balanced as possible, her digestion was gradually re-established, her blood tissue rebuilt, and her nerve system soon in better working order. Then a generous system of dieting, with exercise in the open air, taken systematically, steadily improved her condition and re-established her health. By degrees her muscular strength was regained, and much of the *embonpoint* of her youth restored.

Her *phantom* vanished, never to return, and within a few months she was able to resume her place in the social world, and follow up again her literary pursuits. Her health and strength were sufficiently restored by the first of the following May (five months) to enable her to make a trip picnicing to the Stone Mountain, and climb without aid to its summit and return. Her health continued good, except an occasional feeling of depression from too sedentary habits and over-mental tax, until the date of her death, from another cause*

*An attack of epidemic dysentery—"bloody flux"—in the latter part of the fall of 1874, proved fatal to her. She was taken ill with it while absent on a visit in a distant part of the State; in which condition she was brought to this city, and did not long survive. Up to this time, no return of her

In the rapid strides made by our science in these latter years, there is no department in which progress has been more markedly made than in that pertaining peculiarly to the gentler sex. Yet apace with this progress come dangers to which I must be allowed to allude; and the case just reported, with its extended history, from the pen of the patient herself, will serve to point these allusions and illustrate the same from a feeling standpoint.

In this progress of our science, a few leading minds in different parts of the world have led out into this field and given the results of their observation and experience to the profession. These leaders have been men endowed in every respect with a thorough knowledge of our science in all its departments—with its fundamental principles, its history, its theories, and the results of practice and experience of others. With this store of knowledge to bear with them, they have entered this special department and cultivated this new field to the honor of themselves and their country, sending their name and their fame throughout the world. This success has stimulated others equally prepared to enter the same field, and they too have cultivated with eminent success. But in giving to the world the results of their extended experience, they have as a general rule, to too great an extent, unfortunately neglected to reiterate the great *principles* they carried with them into their new field of labor—*principles* that belong to our common science, and *principles* that should be carried into every special department. The success of these two leading classes, in this as in other special departments of medicine, stimulate still others, whose ambition leads them to enter by some nearer route—to scale the walls, as it were—*to leave these great fundamental principles*, as prosy non-essentials, too tedious and time-requiring with which to allow their ambition to be retarded. They set out, therefore, to enter these fields by some flank movement; they skim over anatomy and ignore histology; they regard physiology as a pretty theory and pathology as a speculation, but neither suited to their tastes; they leave chemistry to the alchemist, and regard ma-

---

former troubles had presented themselves. Fully aware that the end was at hand, she, like a Christian philosopher, as she was, coolly and calmly bade her friends and the world farewell, and quietly passed "beyond the River."

teria medica and therapeutics as an *ollapodrida* with which they are not concerned further than to learn the classification and doses of a few medicines. Their aim and object is special work and at once.

Another class who appear in these special fields have once, more carefully cultivated these principles, but having entered for special work, leave these behind, and as surely become circumscribed in their views, narrowed in their observations, and contracted in their physiological and pathological reasoning and their therapeutic applications. The result is, with both classes, that by two routes they arrive at the same point. The one who scales the walls, or enters the field by flank movements, is ever in search of a special and specific remedy, and the other lapses into methods in treatment, and both into regular routinism; both advancing backwards by the century to the methodic medicine of the Galenical school. They narrow themselves down, in the gynæcological field, into the idea that a woman consists in a uterus and its appendages, and outside of this their is little worthy of their *special* attention. Their *armamentarium medicorum*, or *gynœcologicum*, if you please, consists of a speculum and a porte caustic, with which they expect to battle all the ills peculiar to the sex, as has been so forcibly elucidated in the report of the case herewith given.

It is high time that the regular profession take a bold, outspoken stand against these things, and bring at least to the light of that profession the practices of these intinerent pretenders, these professional Bohemians, who have nothing to lose, and with which this country is above all others infested. It is true that they will cry "persecution," and take advantage of this to play upon the sympathies and gullability of the people while they ply their vocation. They seek notoriety, as their placards and dodgers, their newspaper notices and the results clearly prove. Yet this should not deter the regular profession from at least an effort to save their neighbors from the merciless clutches of a set of roving practitioners, whom fate seems somehow to have foisted upon the profession, as Juergensen would express it, for the special purpose of punishing their fellow-creatures.

If a man desires to enter a special field and cultivate it, let him enter it legitimately, and cultivate it faithfully. Not only this, but let him carry with him into these special departments, for his government and guidance there, the great *principles* inculcated in his professional education. Such a course will prove a benefit to mankind and an honor to the profession, and serve to advance the science of medicine in its every department.

And now, if in conclusion, the report of this case shall in any measure serve as a plea for PRINCIPLES and CONSERVATISM in the treatment of diseases peculiar to the gentler sex, the writer will feel that he is in a much greater measure repaid for not only the time expended in hurriedly penning these lines, but for the many anxious hours of arduous labor passed in striving to study THESE PRINCIPLES and inculcate THIS CONSERVATISM.

99 *Walton Street, July 1st*, 1876.

